# Voluntary wheel running prevented the short-term behavioural effects of vicarious intermittent social defeat in female mice

**DOI:** 10.1007/s00213-025-06927-3

**Published:** 2025-10-10

**Authors:** María Ángeles Martínez-Caballero, Claudia Calpe-López, María Pilar García-Pardo, María Carmen Arenas, Carmen Manzanedo, María Asunción Aguilar

**Affiliations:** 1 Neurobehavioural Mechanisms and Endophenotypes of Addictive Behaviour Research Unit, Department of Psychobiology, University of Valencia, Valencia, Spain; 2 Institute of Psychopharmacology, Central Institute of Mental Health, Medical Faculty Mannheim, University of Heidelberg, Mannheim, Germany; 3 Department of Psychology and Sociology, Faculty of Social Sciences, University of Zaragoza, Teruel, Spain; 4 Department of Psychobiology, University of Valencia, Valencia, Spain

**Keywords:** Anxiety, Depression, Cocaine, Female, Recognition memory, Exercise, Social stress

## Abstract

In a previous study, we observed that a Vicarious Intermittent Social Defeat (VISD) protocol induced anxiety- and depression-like symptoms in late adolescent female mice, and in some of them, enhanced sensitivity to cocaine reward in adulthood. Exposure to voluntary physical activity has been shown to mitigate the effects of different stress protocols in both male and female mice. Therefore, the aim of this study was to evaluate the efficacy of a voluntary wheel running (VWR) procedure in preventing the effects of the VISD in female mice. Four groups of female mice were used; (1) mice without exercise (control) nor stress (only exploration of an empty cage) (CONTROL + EXPL); (2) mice without exercise and exposed to VISD on PND 47, 50, 53 and 56 (CONTROL + VISD), (3) mice exposed to VWR (1 h, 3 days/week, from PND 21 to PND 46) without stress (VWR + EXPL); (4) mice exposed to both VWR and VISD (VWR + VISD). On PND 57–58, all female mice performed the Elevated Plus Maze, Hole-Board, Social Interaction, Splash and Object Recognition tests. Three weeks later, all female mice underwent a place conditioning procedure with cocaine. VWR exposure attenuated the VISD-induced reduction in the percentage of time spent in the open arms of the elevated plus maze and prevented the effects of the VISD in the hole-board, splash and object recognition tests. In addition, VWR facilitated the acquisition of the cocaine-induced conditioned place preference. Therefore, access to exercise during adolescence induced protective effects against the short-term negative consequences of social stress in female mice.

**Supplementary Information:**

The online version contains supplementary material available at 10.1007/s00213-025-06927-3.

## Introduction

Chronic stress has been identified as a contributing factor to the development of mental and substance use disorders (Bommaraju et al. 2024; Hinkelmann and Rose 2025; Sinha 2024). Individuals with anxiety and depressive disorders are more likely to use cocaine (Turner et al. 2018) and the exposure to stressful events can facilitate the transition to cocaine dependence and relapse after abstinence (Sinha 2024). In a previous study of our laboratory, we observed that the exposure to a protocol of Vicarious Intermittent Social Defeat (VISD) during late adolescence was effective to induce stress in female mice, which displayed anxiety- and depression-like symptoms, increased novelty-seeking behaviour and improved recognition memory; besides, some female mice more susceptible to the effects of VISD also showed a long-term enhancement in the vulnerability to the rewarding effects of cocaine in the conditioned place preference (CPP) paradigm (Martínez-Caballero et al. 2024).

The development of strategies to prevent the effects of stress has become a significant area of interest and can be particularly relevant in the context of cocaine addiction, given the absence of approved pharmacological treatments for this disorder. A notable body of research has examined the efficacy of environmental strategies, such as the exposure to physical activity, in promoting resilience to the effects of stress. In this line, a protocol of voluntary wheel running has been shown to prevent the anxiety- and depressive-like symptoms induced by the exposure to social defeat in male mice (Calpe-López et al. 2022) as well as the potentiation of cocaine CPP in defeated male mice (Calpe-López et al. 2022; Ferrer-Pérez et al. 2022). Positive effects of the exercise have also been observed in female rodents; those exposed to several stress protocols with access to a running wheel showed a reduction of depressive- and anxiety-like symptoms compared with sedentary animals (Elias et al. 2023; Robinson et al. 2019; Watanasriyakul et al. 2018). In addition, physical activity prevented the reduction of social interaction observed in sedentary female rats exposed to an uncontrollable tail shock (Tanner et al. 2019, 2023). Moreover, exercising rats showed reduced acquisition of cocaine self-administration and lower responses during extinction and reinstatement tests when compared to sedentary female rats (Smith et al. 2011, 2012; Zlebnik and Carroll 2015). However, no work has hitherto studied the influence of physical activity in reversing the effects of the stress induced by social defeat in female mice.

As mentioned before, VISD induced a complex pattern of behavioural effects including one of a positive nature (enhanced memory in the object recognition test), but mostly of a negative nature, such as anxiety- and depression-like symptoms in the elevated plus maze and splash test, respectively; increased novelty-seeking behaviour in the hole-board test; and, in some vulnerable mice, a deficit of social interaction and enhanced sensitivity to cocaine CPP (Martínez-Caballero et al. 2024). Therefore, the present study sought to evaluate whether voluntary wheel running has protective effects against the short- and long-term consequences of the VISD protocol in female mice. Our hypothesis was that exercise would prevent all the effects of VISD, irrespective of their positive or negative nature. Since some sex differences have been observed in the behavioural profile of mice that were resilient to the effects of social defeat (Calpe-López et al. 2020; Martínez-Caballero et al. 2024), the novelty of the present work lies not only in studying the effects of physical activity in females exposed to VISD but also in evaluating whether there are differences between the pro-resilient effects of exercise on the consequences of social stress in male and female mice.

## Methods and materials

### Subjects

A total of 79 mice (51 female and 28 male) of the C57BL/6J strain and 28 male mice of the OF1 strain were supplied by Charles River (France) and arrived at the laboratory on post-natal day (PND) 42. C57BL/6J mice were housed in groups (3–4 mice) in plastic cages (25 × 25 × 14.5 cm) for a period of one week before the initiation of the experimental procedures. OF1 male mice were housed individually in plastic cages (23 × 32 × 20 cm) in order to induce aggressiveness (Rodríguez-Arias et al. 1998). All mice were housed in conditions of constant temperature (21 ± 1 °C), a reversed light schedule (white light from 19:30 to 07:30) and food (standard chow) and water available *ad libitum* (except during behavioural tests). All experimental protocols were conducted in accordance with Directive 210/63/EU and were approved by the Ethics Committee in Experimental Research of the University of Valencia (A20240214124843, 2024-VSC-PEA-0079).

### Drugs

The experimental female mice were injected intraperitoneally with 1.5 mg/kg of cocaine (Alcaliber Laboratory, Madrid, Spain), dissolved in physiological saline (NaCI 0.9%) to a volume of 0.01 ml/g of body weight.

### Experimental protocols

#### Voluntary wheel running (VWR)

Eight low-profile running wheels (Med Associates Inc.) were employed. Each wheel is made of plastic (10.25 × 15.5 × 13.7 cm) and rotates on a central axis in a horizontal plane allowing physical activity through spontaneous locomotion. On PND 25–46, female mice in the voluntary wheel running (VWR) condition were individually placed in a plastic cage that contained a running wheel, three times per week (Monday, Wednesday and Friday) for one hour. During this time (a total of 11 sessions of 1 h) mice can voluntarily engage in physical activity. Mice in the control condition (no exercise) were placed in the same plastic cages but without any wheel. All sessions were carried out in different rounds between 9.00 h and 15.00 h, with the mice being exposed to VWR in different rounds every day (mice in the first round on day 1 were exposed to the wheel in the second round on day 2, etc.). The procedure, timing and duration of this VWR protocol were based on the previous work of our laboratory (Calpe-López et al. 2022).

#### Vicarious intermittent social defeat (VISD)

To induce social stress in the female mice, they were exposed to four episodes of vicarious defeat separated by intervals of 72 h (PND 47, 50, 53 and 56), based on the protocol of VISD previously used (Martínez-Caballero et al. 2024). During each episode, an experimental C57BL/6J female mouse was placed in the cage of an OF1 male mouse for five minutes and, protected by a perforated methacrylate wall, perceived through non-physical (visual, olfactory and chemosensory) stimuli how a C57BL/6J male mouse was defeated by the resident mouse. In a previous study we have demonstrated that this protocol was effective to induce physiological and behavioural markers of stress in female mice (Martínez-Caballero et al. 2024). The groups not exposed to stress underwent the same protocol but the female mouse was placed in an empty cage (without a resident male mouse) and only performed exploration (Expl) of the cage. Further details about the protocol can be found in Supplementary Material.

### Experimental design and behavioural tests

**Fig. 1:**
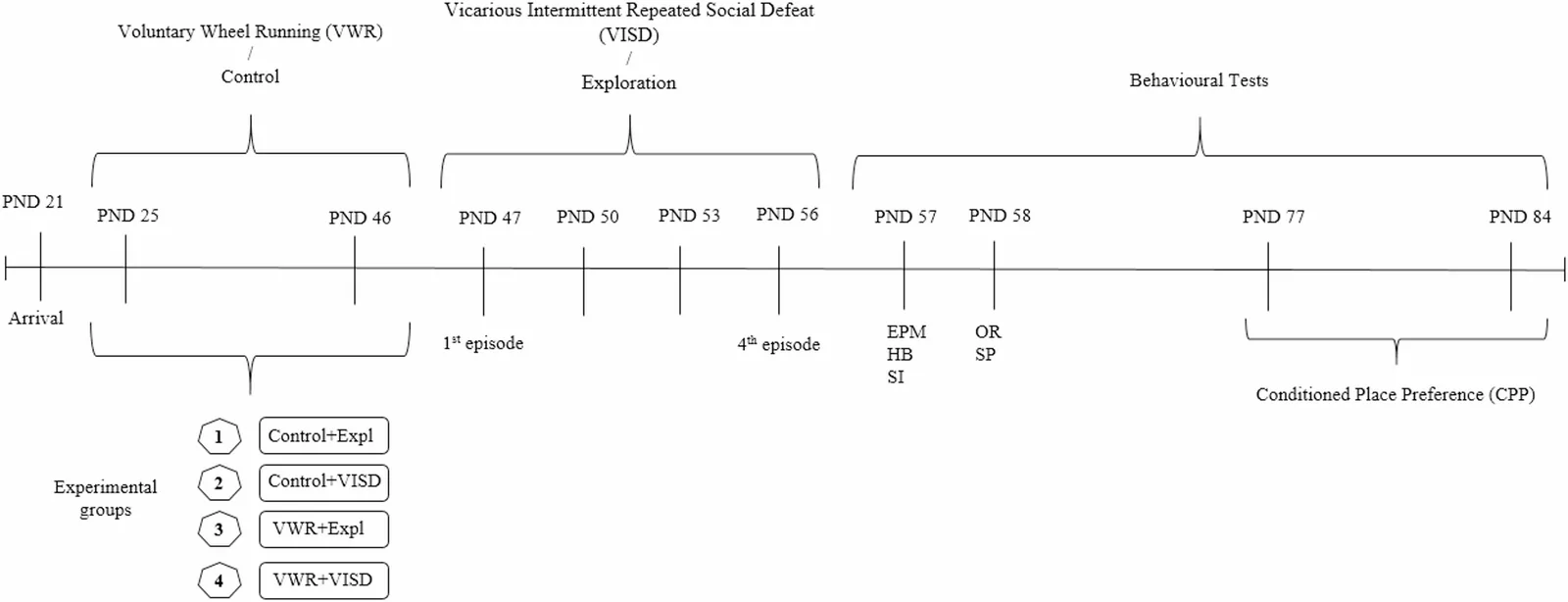
Experimental design. EPM, Elevated plus maze; HB, Hole-Board test; SI, Social Interaction test; OR, Object recognition test; SP, Splash test

The experimental C57BL/6 female mice were assigned to four groups: a control group without exercise nor stress (Control + Expl; *n =* 12), a group without exercise and exposed to stress (Control + VISD; *n =* 12*)*, a group with access to voluntary wheel running but without exposure to stress (VWR + Expl; *n* = 11) and a group with access to voluntary wheel running and exposed to stress (VWR + VISD; *n* = 16). In the last group we used a larger sample size in order to observe possible individual differences in the response of stressed female mice to exercise. During adolescence (PND 25–46), the female mice were exposed to exercise or to a cage without running wheel. After this period the animals were no longer exposed to the activity wheels. Subsequently, on PND 47–56 female mice underwent the VISD protocol or the exploration of an empty cage (non-stress). After the last episode of vicarious defeat or exploration, all female mice underwent behavioural testing (Fig. 1). On PND 57, the behaviour of female mice was evaluated in the Elevated plus maze (EPM), Hole-Board and Social Interaction. The EPM, based on the natural aversion of mice to open elevated areas, evaluates anxiety-like symptoms. The time, entries and latency of entry in the open and closed arms and the time and entries in the central platform were measured. Anxiety levels are considered to be higher when the measurements in the open arms are lower. The hole-board evaluates novelty-seeking according to the frequency of dips (introduction of the mouse’s head in a hole) and the latency to perform this behaviour. The social Interaction test evaluates the social behaviour of the female mouse with a conspecific male mouse by calculating an index of social interaction (ISI) that is commonly used as a social preference or social avoidance index. On PND 58, female mice performed the Object recognition test and the Splash test. The Object recognition test evaluates explicit memory by measuring the time that the mouse spent exploring a novel object in comparison with a familiar one (discrimination index). In the Splash test a sucrose solution is sprayed onto the mouse to stimulate grooming behaviour. A reduction in the frequency or time spent in this behaviour or an increase in its latency is interpreted as depression-like behaviour. The order of tests was based on a previous study carried out in our laboratory (Martínez-Caballero et al. 2024), according to the degree of stress that the tests had induced in the mice; in this way, we hoped to prevent previous experience in a test from affecting the performance in subsequent tests. As the open arms measurements are very sensitive to environmental conditions and prior manipulation of the animal, we decided to perform the EPM first. The remaining tests were performed in the order described above, with an interval of at least one hour between each test. After a period of three weeks, the place conditioning procedure was conducted (PND 77–84) to evaluate the long-term effects of the experimental manipulations undergone during adolescence (VWR and VISD) on the acquisition of CPP after conditioning with a low dose of cocaine (1.5 mg/kg), which is not effective to induce CPP in non-stressed female mice (Martínez-Caballero et al. 2024). Further details about the apparatuses, protocols and behavioural measurements can be found in Supplementary material.

### Statistical analysis

Data were screened for outliers using the Z-scores method (Z=(X-mean)/standard deviation) and values with z-scores of ± 2) are considered outliers and removed from datasets used for further statistical analysis (see Supplementary Material). Sample distributions were assessed for normality (Kolmogorov-Smirnov test) and homogeneity (Levene’s test). The short-term effects of exercise and social stress in the behavioural tests were evaluated using a two-way analysis of variance (ANOVA) with two between-subjects variables -Exercise condition- with two levels (Control and VWR) and –Stress- with two levels (Expl and VISD). To evaluate the long-term effects of exercise and social stress on the cocaine CPP, a three-way repeated measures ANOVA with the two between-subjects variables described above and a within-subjects variable –Days- with two levels (Pre-C and Post-C) were used. Post-hoc comparisons were performed with Bonferroni tests. In the case of behavioural measures with unequal variance, one-way Welch’s ANOVA (with the variable Group, with four levels: Control + Expl, Control + VISD, VWR + Expl and VWR + VISD) and Games-Howell post-hoc tests were also used. All statistical analyses were conducted using the SPSS programme.

## Results

### Short-term effects of exercise and vicarious defeat

**Fig. 2:**
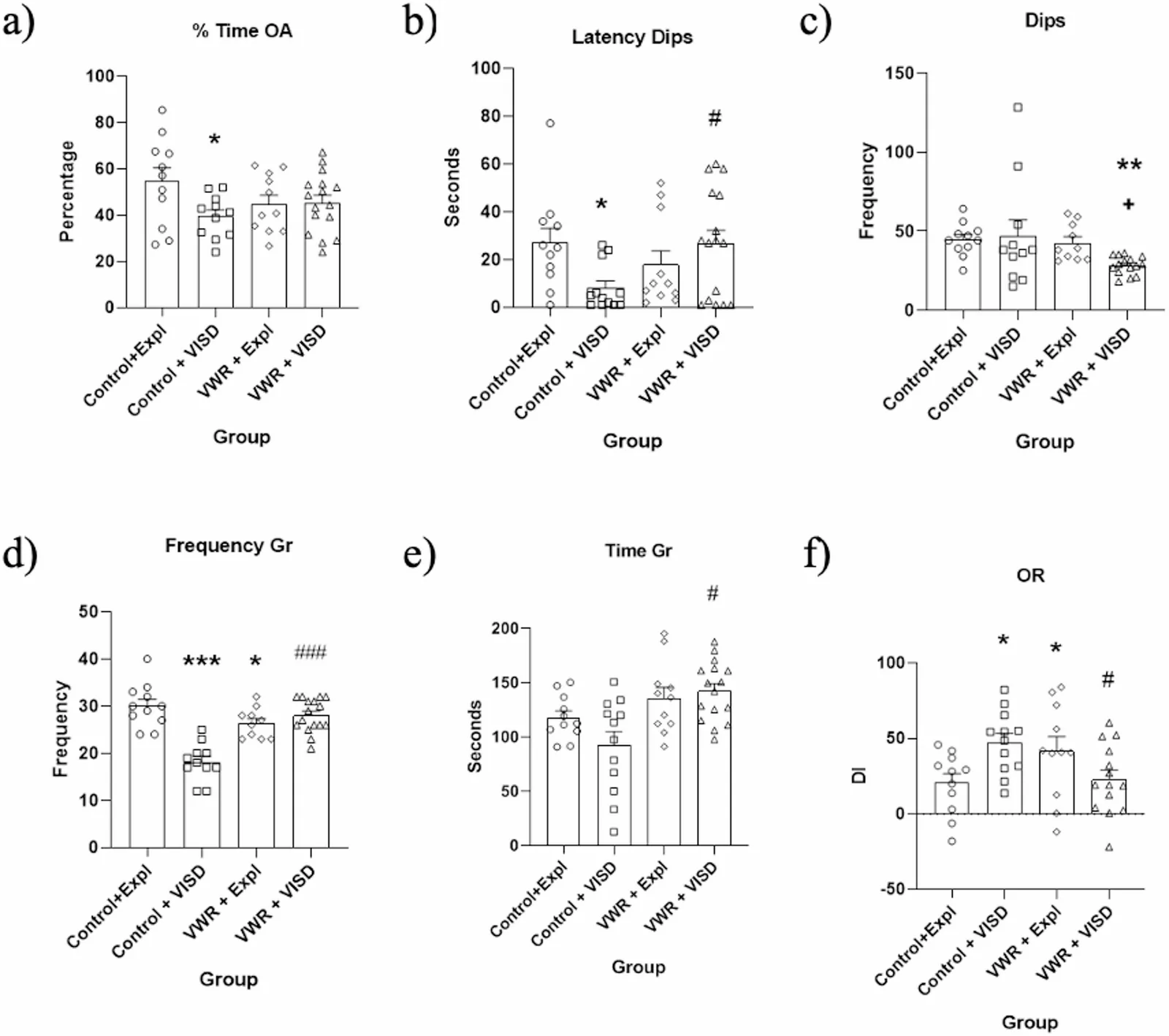
Short-term effects of VISD and VWR in the behavioural tests. Bars represent mean (± SEM) values of the percentage of time spent in the open arms of the EPM (% Time OA) (**a**), the latency in seconds to perform the first dip (**b**) and the frequency of dips (**c**) in the hole-board, the frequency of grooming (Gr) (**d**) and the time spent in this behaviour (**e**) in the splash test and the discrimination index (DI) in the object recognition (OR) test (f). *p < 0.05, **p < 0.01 and ***p < 0.001, significant difference with respect to the Control + Expl group; #*p* < 0.05 and ###*p* < 0.001, significant difference with respect to the Control + VISD group; +*p* < 0.05, significant difference with respect to the VWR + Expl group

In relation to the EPM, two-way ANOVA with data of the percentage of the time spent in open arms (Fig. 2a) revealed that only the Interaction of the variables Stress X Exercise was significant [F(1,45) = 3.911; *p* < 0.05]. Post-hoc comparisons revealed that among the groups of female mice without exercise only mice exposed to vicarious defeat exhibited a lower percentage of time in open arms compared to the non-stressed mice (Control + VISD vs. Control + Expl, *p* < 0.05). Two-way ANOVAs of the other measurements registered in the EPM also showed some significant effects of the variable Stress, Exercise and/or the Interaction Stress X Exercise (see Table 1). Additional Welch’s ANOVAs were performed for data regarding the time spent in open arms, latency to enter the closed arms and percentage of entries in the open arms, since Levene tests showed that these datasets displayed unequal variances across groups. Welch’s ANOVA indicated a significant effect of the variable Group with respect to latency to enter the closed arms [F(3,22) = 3.170; *p* < 0.05], but Post-hoc Games-Howell tests did not reveal any significant differences between the groups.

**Table 1:**
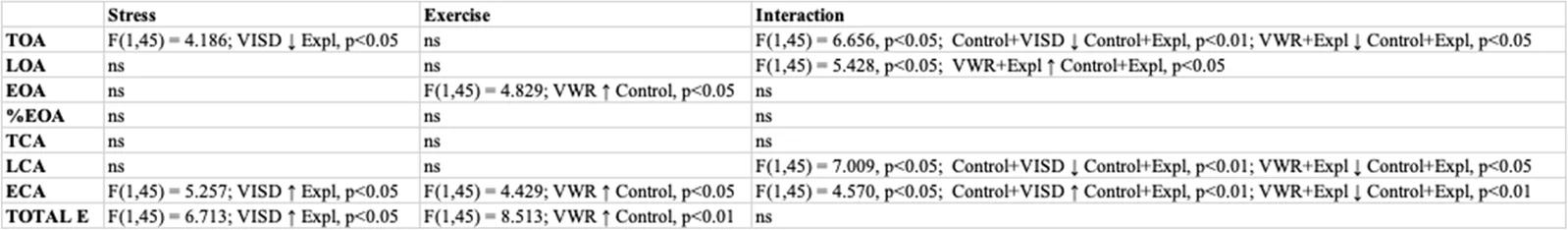
Two-way ANOVAs of the data relative to the following measures of the EPM: time spent in open arms (TOA), latency to enter into the open arms (LOA), entries into the open arms (EOA), percentage of entries into the open arms (%EOA), time spent in closed arms (TCA), latency to enter into the closed arms (LCA), entries into the closed arms (ECA), total entries into the arms (TOTAL E). Post-hoc comparison were performed with bonferroni tests

Two-way ANOVA of the latency to perform the first dip in the hole-board test (Fig. 2b) revealed that only the Interaction Stress X Exercise was significant [F(1,46) = 6.437; *p* < 0.05]. Post-hoc comparisons showed that among the non-exercising mice, those exposed to vicarious defeat displayed a lower latency of dips than those without stress (Control + VISD vs. Control + Expl group, *p* < 0.05). In addition, exercising mice exposed to vicarious defeat showed higher latency of dips than non-exercising stressed mice (VWR + VISD vs. Control + VISD groups, *p* < 0.05), suggesting that exercise prevent the effects of stress. Two-way ANOVA of the frequency of dips did not reveal significant effects. However, Levene’s test showed that this dataset presented unequal variances across groups, while the Welch’s ANOVA indicated a significant effect of the variable Group [F(3,19) = 9.979; *p* < 0.001]. Post-hoc Games-Howell tests showed that the group VWR + VISD performed less dips than the Control + Expl (*p* < 0.002) and the VWR + Expl (*p* < 0.05) groups (see Fig. 2c).

In relation to the splash test, two-way ANOVA of the frequency of grooming (Fig. 2d) revealed that the variables Stress [F(1,44) = 20.645; *p* < 0.001] and Exercise [F(1,44) = 7.534; *p* < 0.01] as well as the Interaction Stress X Exercise [F(1,44) = 36.218; *p* < 0.001] were significant. Post-hoc comparisons of the variable Stress demonstrated that stressed mice exhibited a lower frequency of grooming than non-stressed mice (*p* < 0.001) while post-hoc comparison of the variable Exercise showed that mice exposed to exercise displayed a higher frequency of this behaviour than control mice (*p* < 0.01). In addition, post-hoc comparisons of the Interaction revealed that the groups exposed only to vicarious defeat (Control + VISD) or wheel running (VWR + Expl) exhibited a lower frequency of grooming in comparison to the Control + Expl group (*p* < 0.001 and *p* < 0.05, respectively). Furthermore, the group exposed to exercise plus vicarious defeat demonstrated a higher frequency of grooming than the group exposed only to vicarious defeat (VWR + VISD vs. Control + VISD groups, *p* < 0.001), suggesting that exercise prevent the effects of stress. Regarding the time spent in grooming, two-way ANOVA showed that only the variable Exercise was significant [F(1,46) = 13.612; *p* < 0.001]; mice exposed to running spent more time in grooming behaviour than control mice. Levene test showed that this dataset showed unequal variances across groups and the additional Welch’s ANOVA indicated a significant effect of the variable Group [F(3,24) = 4.872; *p* < 0.01]. Post-hoc Games-Howell tests showed that the group VWR + VISD spent more time in grooming than the group Control + VISD (see Fig. 2e). Two-way ANOVA of the latency of grooming (and the additional Welch’s ANOVA required for unequal variances) did not reveal significant effects.

With regard to the object recognition test, the two-way ANOVA of the discrimination index (Fig. 2f) revealed that the Interaction Stress X Exercise was significant [F(1,44) = 10.569; *p* < 0.01]. Post-hoc comparisons confirmed that the groups exposed only to vicarious defeat (Control + VISD) or wheel running (VWR + Expl) showed a higher discrimination index than the Control + Expl group (ps < 0.05). Furthermore, the group exposed to exercise plus vicarious defeat demonstrated a lower discrimination index than the group exposed only to vicarious defeat (VWR + VISD vs. Control + VISD groups, *p* < 0.05), suggesting that exercise prevent the effects of stress.

Finally, two-way ANOVA of the social interaction index did not reveal significant effects of the variable Stress [F(1,44) = 0.222; *p* = 0.640], Exercise [F(1,44) = 2.127; *p* = 0.152] or the Interaction Stress X Exercise [F(1,44) = 0.710; *p* = 0.404] (see Figure S1 in the Supplementary Material).

### Long-term effects of exercise and vicarious defeat on the cocaine CPP

**Fig. 3:**
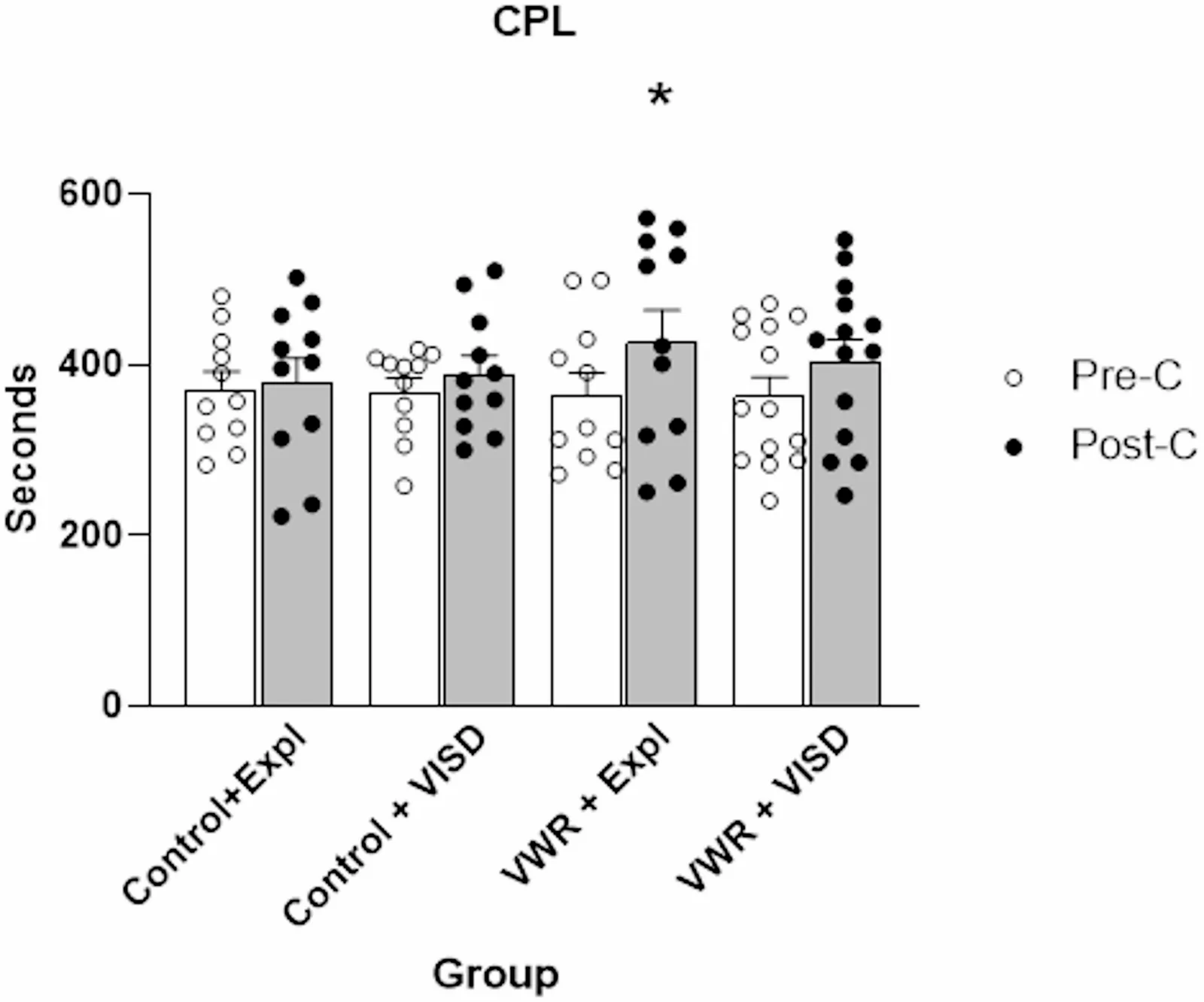
Effects of VWR and VISD in the cocaine CPP. Bars represent mean (± SEM) values of the time (in seconds) spent in the drug-paired compartment in the Pre-C and Post-C. *p < 0.05, significant difference Post-C vs. Pre-C of the same group

ANOVA of the CPP data revealed that only the variable Days was significant [F(1,43) = 7.846; *p* < 0.01], with mice spending more time in the drug-paired compartment in Post-C than in Pre-C (Fig. 3). Multivariate Simple Effects of this variable demonstrated that the effect of Days [F(1,44) = 6.562; *p* < 0.05] was significant in the group exposed only to exercise (VWR + Expl). Bonferroni test revealed that only the mice in this group spent more time in the drug-paired compartment in Post-C in comparison to Pre-C (*p* < 0.05), suggesting the acquisition of CPP.

## Discussion

As expected, our protocol of VISD induced a complex pattern of short-term behavioural effects, including anxiety- and depressive-like symptoms, increased novelty-seeking behaviour and enhanced recognition memory. Voluntary exercise during adolescence was effective in attenuating or completely preventing most of these effects of stress. Furthermore, the exposure to physical activity during adolescence facilitated the acquisition of cocaine CPP in adulthood. These results indicate that access to exercise during adolescence can induce beneficial effects, protecting female mice against some of the negative short-term effects of social stress, such as the development of depression-like symptoms and the enhancement of novelty-seeking behaviour. However, exercise can also induce anxiogenic effects and reverse the positive effects of social stress on recognition memory.

### Short-term behavioural effects of exercise and vicarious defeat

In the present study, late adolescent female mice exposed to VISD showed anxiety-like symptoms, as indicated by the reduced percentage of time spent in the open arms of the EPM in comparison with female mice not exposed to stress. This effect was previously observed in another study conducted in our laboratory with the same protocol of VISD (Martínez-Caballero et al. 2024). Exposure to exercise during adolescence seems to have negligible effects on the anxiety-like behaviour induced by this stress protocol, as the mice exposed to both wheel running and vicarious defeat did not differ from non-stressed control mice or from mice exposed only to stress. The influence of exercise on the anxiety-like symptoms induced by VISD has not been previously studied; however, research with other stress protocols has demonstrated protective effects. A high time or percentage of time in the open arms of the EPM was observed in groups of female rodents that performed exercise and were exposed to isolation and chronic mild stress or to the combination of multiple stressors in a day (Robinson et al. 2019; Watanasriyakul et al. 2018). The more limited effects observed in our study can be explained by the period in which the female rodent could access the exercise. While in the present work the VWR was performed during early adolescence until VISD exposure, in the other studies the access to VWR was limited to the period of stress (Watanasriyakul et al. 2018) or permitted after exposure to stress (Robinson et al. 2019). This hypothesis is further corroborated by a recent study which found no differences in the EPM between female rats subjected to chronic restraint stress and those given access to wheel running several weeks before the stress (Williams et al. 2023). It is noteworthy that we also observed that vicarious defeat and physical activity induced similar effects in other measures of the EPM, for example, both protocols increased the entries into the closed arms and the total entries into the arms (that suggests an increase in the motor activity). As commented above, the anxiogenic effects of VIDS in the EPM have been previously observed (Martínez-Caballero et al. 2024), but exercise did not induce anxiety-like behaviour in the EPM in female mice without stress exposure (Rauhut et al. 2024; Williams et al. 2023). However, in agreement with our results, female mice exposed to voluntary exercise displayed anxiety-like behaviour in the open field test (Rauhut et al. 2024) and the same effect was observed in male mice in the EPM (Pan-Vazquez et al. 2015; Calpe-López et al. 2022) and other tests of anxiety (Fuss et al. 2010a, b). Another fact that may have contributed to the anxiety-like effects observed in the VWR + Expl groups is the cessation of exercise, which might have induced a withdrawal-like state. It has been observed that forced cessation of voluntary wheel running after 6 weeks of continuous access to exercise induces anxiety-like effects in male rats (Greenwood et al. 2012). In addition, using the same schedule of voluntary wheel running and behavioural testing as in the present study, we previously observed anxiety-like effects in male mice exposed to exercise. However, it is important to note that, in contrast to that observed herein, voluntary wheel running reversed the anxiety-like effects induced by social defeat (Calpe-López et al. 2022). Thus, sex differences can be observed in the protective effects of exercise on anxiety-like behaviour induced by social stress.

With regard to the hole-board test, as was demonstrated in a previous study (Martínez-Caballero et al. 2024), female mice exposed to VISD showed a lower latency to perform the first dip in comparison to non-stressed mice, which can be interpreted as an increase in the novelty-seeking behaviour. The novelty-seeking trait has been associated with vulnerability to drug addiction in humans and animals (Arenas et al. 2016; Wingo et al. 2016). For example, adolescent female mice with higher novelty-seeking are more sensitive to the rewarding effects of cocaine in the CPP paradigm (Arenas et al. 2014). In the case of stressed female mice, we have previously observed that mice with lower latency of dips develop CPP with a subthreshold dose of cocaine (Martínez-Caballero et al. 2024, 2025). Thus, the fact that VISD increases novelty-seeking could be interpreted as a negative behavioural effect that makes female mice more vulnerable to cocaine reward. Exposure to exercise did not induce effects by itself, but was effective in preventing the effect of VISD on latency of dips. In addition, mice in the VWR + VISD group performed less dips than non-stressed mice (Control + Expl and VWR + Expl groups). These results suggest that VWR during adolescence can reduces novelty-seeking in stressed female mice and, consequently, their vulnerability to cocaine. To the best of our knowledge, the influence of exercise on stress-induced increase in novelty-seeking behaviour in female mice has not been previously studied; however, it has been observed that male mice exposed to intermittent social defeat stress showed low novelty-seeking behaviour in the hole-board test, independent of exposure or not to exercise (Calpe-López et al. 2022). These divergent results in female and male mice underscore the critical role of sex in the impact of stress on novelty-seeking behaviour and its modulation by physical activity.

VISD also induced depressive-like symptoms in the splash test, as evidenced by the reduction of grooming behaviour, in agreement with our previous results (Martínez-Caballero et al. 2024). These effects were blunted in stressed female mice with access to exercise (VWR + VISD group), who showed levels of grooming behaviour similar to non-stressed mice. This suggests that exercise can effectively prevent depressive-like symptoms induced by social stress. A similar effect was observed in female mice exposed to an unpredictable chronic mild stress paradigm. Stressed females with access to running wheels showed increased sucrose consumption and reduced immobility in the forced swim and tail suspension tests compared to those without this exercise condition (Elias et al. 2023). In addition, female rats exposed to isolation plus chronic mild stress with access to exercise showed lower immobility in the forced swim and tail suspension tests compared to stressed females that were sedentary (Watanasriyakul et al. 2018). It is important to note that although we observed that mice exposed only to voluntary wheel running showed a slight reduction in the frequency of grooming, this result does not indicate the presence of depression-like symptoms in mice exposed to exercise, which, in fact, spent more time in this behaviour. Regarding the influence of sex, in our previous study using the same protocol of voluntary wheel running in male mice, we also found a protective action of exercise on the depression-like effects of social defeat in the splash test.

Our VISD protocol did not reduce social interaction, an effect that could be expected given that the stressor has a strong social component. In fact, it has been reported that female mice exposed to vicarious defeat for ten consecutive days show a deficit of social interaction (Iñiguez et al. 2018; Pagliusi et al. 2022; Morais-Silva et al. 2023). However, in line with the present results, previous studies have observed that intermittent exposure to vicarious defeat did not induce effects in the social interaction test (Ródenas-González et al. 2023; Martínez-Caballero et al. 2024). Such divergences among studies are probably due to the different levels of stress induced by ten daily vs. four intermittent episodes of vicarious defeat. Similarly, exposure to exercise alone or in combination with VISD did not affect levels of social interaction. The lack of effect of VISD on social interaction meant we could not evaluate the potential protective effect of exercise on stress-induced social avoidance, which we have previously observed in male mice (Calpe-López et al. 2022). A recent study has also demonstrated that voluntary wheel running prevents the reduction in juvenile social exploration induced by inescapable tail shock in male and female rats, suggesting that exercise can protect against stress-induced social avoidance in both sexes; however, female rats were more responsive than males to the protective effects of voluntary wheel running against the fear (freezing behaviour) induced by inescapable stress (Tanner et al. 2023).

With regard to the object recognition test, all groups demonstrated an intact recognition memory as indicated by the positive discrimination index (see Supplementary Material). In addition, exposure to VISD enhanced the discrimination index, as previously demonstrated (Martínez-Caballero et al. 2024), i.e. facilitated recognition memory. Although this result may be surprising, it is important to note that chronic stress induces sex-dependent changes in cognition, impairing performance of several cognitive tasks in male rodents without affecting or improving female performance (for a review, see Bowman et al. 2022). An improving effect of recognition memory was also observed in the group exposed to exercise, in line with results of a study employing various running wheel protocols which has reported that female mice subjected to moderate-intensity training exhibited a higher discrimination index than sedentary or high-intensity training females (Feter et al. 2019). More important, we observed that the group exposed to exercise plus stress showed discrimination index values like those of the control non-stressed group. This finding aligns with observations during the other behavioural tests (i.e., the hole-board and splash test) where exposure to voluntary wheel running was able to counteract the impact of VISD, thereby suggesting that exercise can prevent both the negative and positive effects of social stress. Although the efficacy of the exercise in preventing the effect of VISD on recognition memory has not been previously studied, no differences were observed in the recognition memory test with a novel odor between acute stressed female rats exposed to exercise or not (Robinson et al. 2019). Conversely, a study has reported that running during adolescence rescued the deficits in object recognition memory induced by maternal separation in male (Neves et al. 2015) and female mice (Wearick-Silva et al. 2017). Divergent results in the influence of exercise on the effects induced by stress on recognition memory could be due to methodological differences between studies (stress and exercise protocols, sex and age of the animals, species used, etc.).

### Long-term effects of exercise and vicarious defeat on the cocaine CPP

In the long term, VISD did not induce effects in the place conditioning protocol in female mice conditioned with cocaine (as observed previously, Martínez-Caballero et al. 2024). Conversely, exposure to voluntary wheel running during adolescence seems to facilitate the acquisition of cocaine CPP. In a similar line, it has been observed that exposure to exercise increases the CPP induced by cocaine in female rats (Smith et al. 2008). In our opinion, this effect does not indicate that exercise increase the vulnerability of female mice to cocaine for two reasons. Firstly, several studies have showed that exposure to physical activity appears to reduce the rewarding effects of cocaine. In comparison to sedentary rats, exercising rats self-administered less cocaine (Smith et al. 2011) and responded less during extinction (Smith et al. 2012) and during cue- and priming-induced reinstatement tests (Smith et al. 2012; Zlebnik and Carroll 2015). In a similar manner, female mice that had access to voluntary wheel running during adolescence exhibited a reduction in cocaine-induced behavioural sensitisation (Lespine and Tirelli 2018; Becker et al. 2021). Secondly, there is evidence that exercise facilitated the acquisition of Pavlovian associations (Baruch et al. 2004). For example, voluntary exercise facilitated both the CPP induced by morphine (Eisenstein and Holmes 2007) and the conditioned place aversion induced by spiradoline (Smith et al. 2004). An alternative explanation for the CPP observed in the VWR + Expl group is that, as previously mentioned, the sudden cessation of exercise may have induced a withdrawal-like state that was manifested by the presence of short-term anxiety-like effects, and also in the long term, manifested as an enhanced sensitivity to the rewarding effects of cocaine. In line with this, Greenwood et al. (2012) observed an increase in anxiety-like behaviour in male rats 25 days after forced cessation of voluntary wheel running.

We also observed that female mice with access to exercise and exposure to stress did not develop cocaine CPP. No prior studies have examined the impact of exercise on the rewarding effects of cocaine in stressed female mice, however, the exposure to voluntary wheel running was found to be effective in preventing the defeat-induced potentiation of cocaine CPP in male mice (Calpe-López et al. 2022; Ferrer-Pérez et al. 2022). In the present study, stress exposure did not affect cocaine reward itself but reversed the cocaine CPP induced by voluntary wheel running. This effect could be due to the impairing effects of stress on pavlovian learning, counteracting the positive effects of exercise in this type of learning. In support of this hypothesis, it has been observed that exposure to social defeat during adolescence impaired fear conditioning in male rats (Novick et al. 2016). The fact that VISD did not increase the rewarding effects of cocaine is a limitation of the present study. Future studies should employ a higher dose of cocaine that, without inducing CPP itself in naïve female mice, can increase cocaine CPP in stressed animals; in this way, the potential protective effects of exercise on the stress-induced potentiation of cocaine reward can be explored.

In conclusion, the fact that stressed female mice exposed to voluntary wheel running showed a resilient profile to some of the short-term effects of vicarious defeat (for example, the absence of depression-like symptoms), indicates that exposure to physical activity during adolescence is a useful environmental strategy to prevent the effects of social stress, as we have previously observed with defeated male mice. However, our study also suggests sex differences in relation to the potential of voluntary wheel running to prevent some of the effects of social stress; for example, exercise prevented anxiety-like behaviour in male, but not in female mice. An interesting paradox is that voluntary wheel running induced similar effects to social defeat in mice without stress exposure, but induced protective effects in stressed mice. It could be hypothesised that voluntary physical activity is a rewarding activity that inoculates against the effects of stress and that the anxiogenic effects observed in non-stressed mice exposed to exercise were due to the forced cessation of voluntary wheel running that induced withdrawal-like effects. Therefore, a lifestyle that incorporates routine physical activity several times a week could help to reduce the negative consequences of social stress, so prevalent in contemporary society.

## Supplementary Information

Below is the link to the electronic supplementary material.

Supplementary Material 1

## Data Availability

Data will be made available on request.
