# Supplementary material for: Voluntary wheel running prevented the short-term behavioural effects of vicarious intermittent social defeat in female mice
Source: Psychopharmacology (Berl). 2025 Oct 10;243(8):1845–55. doi: 10.1007/s00213-025-06927-3 (PMC13506583; doi:10.1007/s00213-025-06927-3)
Supplement: Supplementary file 1 [file 213_2025_6927_MOESM1_ESM.docx]

**SUPPLEMENTARY MATERIAL**

**Experimental Protocols**

*Vicarious Intermittent Social Defeat (VISD)*

The VISD protocol, which was used to induce social stress in female mice (Martínez-Caballero et al., 2024), consisted of four episodes during the post-natal days (PND) 47, 50, 53 and 56 (separated by intervals of 72 hours). During each episode, lasting five minutes, the female mouse was introduced into the home cage of an aggressor male mouse of the OF1 strain (the resident mouse), with both mice separated by a perforated methacrylate wall. Then, a male mouse of the same strain as the experimental female mouse (C57BL/6J intruder) was introduced into the compartment where the resident male was, and a confrontation between the two male mice was carried out. Throughout the four episodes, the intruder mice were exposed to a different resident mouse. All intruder mice displayed avoidance/flee and defense/submissive behaviours given that they faced resident mice with high levels of aggressive (threat and attack) behaviours. The criterion used to define an animal as defeated was the adoption of a specific posture of defeat characterized by upright submissive position, limp forepaws, upwardly angled head, and retracted ears (Miczek et al., 1982; Ribeiro Do Couto et al., 2006). The female mouse paid attention to the interaction between male mice and witnessed how the intruder is defeated by the resident mouse. The use of the perforated methacrylate wall enabled the perception of visual, olfactory and chemosensory (but not physical) stimuli of defeat. The non-stressed female mice performed the same protocol without the presence of an aggressor male mouse.

*Elevated Plus Maze (EPM)*

EPM was conducted on PND 57 to evaluate anxiety-like symptoms (Campos et al., 2013; Rodgers and Johnson, 1995) in an apparatus comprising a central platform (5 x 5 cm) with two open arms and two closed arms (30 x 5 cm each one) that was elevated 45 cm above floor level. Each mouse was placed on the central platform, facing an open arm, and was allowed to explore for five minutes. Each trial was videotaped and the behaviour of the mice was analysed using a computerised method (Raton Time 1.0 software; Fixma SL, Valencia, Spain). The time and percentage of time [(open/open+closed) x 100] spent in the open arms, the latency to enter for the first time, as well as the entries and percentage of entries in open arms were recorded.

*Hole Board (HB) test*

In order to evaluate the novelty-seeking behaviour of the mouse (Arenas et al., 2014), the HB test was performed on PND 57. The experiment was conducted using a square box (28 x 28 x 20.5 cm) with 16 equidistant holes of 3 cm in diameter on the floor and transparent plexiglas walls (CIBERTEC SA, Madrid, Spain). The mouse was introduced into a corner of the box and permitted to explore the cage for a period of 10 minutes. The total number of dips and the latency to perform the first dip were recorded by the apparatus via photocells located beneath the surface of the holes.

*Social Interaction (SI) test*

Twenty-four hours after the last defeat or exploration (PND 57), the social behaviour of the mice was evaluated in an open field (37 × 37 × 30 cm). A perforated plexiglass cage (10 × 6.5 × 30 cm) was placed in the middle of one wall of the open field. Following habituation to the room, each animal was placed in the centre of the open field and permitted to explore it twice, under two different experimental conditions. On the first occasion (the object phase), the perforated plexiglass cage was empty. After 10 minutes of exploration, the experimental mouse was returned to its home cage for 2 minutes. Subsequently, a male mouse of the same strain was confined to the perforated cage and the experimental mouse was reintroduced into the open field for 10 min (social phase).In both phases, the time spent in the 8 cm area surrounding the perforated cage—the interaction zone—was registered and automatically sent to a computer using the Ethovision 2.0 software package (Noldus, Wageningen, The Netherlands). The resultant data was utilised to calculate an index of social interaction (ISI), which is defined as the time spent in the interaction zone during the social phase divided by the total time spent in the interaction zone (both phases) (Henriques-Alves & Queiroz, 2016). This ISI is widely employed as a social preference-avoidance index (Krishnan et al., 2007).

*Splash (SP) test*

The SP test was conducted on PND 58 to evaluate depressive-like symptoms (Smolinsky et al., 2009). In this test, each mouse was placed in a transparent cage (15 x 30 x 20 cm) and sprayed with a 10% sucrose solution on the dorsal coat. The behaviour of the mice was then videotaped and analysed using a computerised method (Raton Time 1.0 software; Fixma SL, Valencia, Spain). The behaviour of the mice was evaluated based on three parameters: time spent in grooming, the latency to the first grooming, and the frequency of grooming.

*Object Recognition (OR) test*

In order to evaluate explicit memory, the OR test was performed (Montesinos et al., 2015). This test consists of three phases: habituation, training session (T1) and test session (T2). During habituation (on PND 57), the mouse is introduced into a plastic cage (25 x 25 x 14.5 cm) and can explore for 2 minutes. On PND 58, T1 and T2 were performed. During T1, the mouse was placed in the plastic cage (25 x 25 x 14.5 cm) for 5 minutes, with two stones placed in two different corners. Subsequently, the mouse returned to the home cage for one minute (memory retention interval). In the T2, the mouse was introduced again in the cage with a stone and a small non-toxic plastic toy for 5 minutes. The total time spent exploring the stone and the toy in T2 was recorded and analysed using a computerised method (Raton Time 1.0 software; Fixma SL, Valencia, Spain) to calculate the discrimination index (DI): [DI = (Tnovel-Tfamiliar)/(Tnovel+Tfamiliar) X 100%].

*Conditioned Place Preference (CPP)*

The CPP procedure was used to evaluate the conditioned rewarding effects of cocaine (Maldonado et al., 2007). The apparatus used consisted of a Plexiglas box with two compartments (30.7 cm long x 31.5 cm wide x 34.5 cm high each one) with different coloured walls (white vs. black) and floor textures (wide grid vs fine grid). Both compartments are separated by a grey central area (13.8 cm long x 31.5 cm wide x 34.5 cm high), and the position of the mouse was recorded by four infrared light beams in each compartment and six in the central area. The equipment was controlled by three IBM PC computers using MONPRE 2Z software (Cibertec SA, Madrid, Spain). The CPP procedure was carried out in three phases: Pre-Conditioning (Pre-C), Conditioning and Post-Conditioning (Post-C). During the Pre-C, the mouse explored the box for 15 minutes and the time spent in each compartment was recorded. Mice that showed a strong unconditioned aversion or a preference for a specific compartment were excluded from the study. In the conditioning phase, mice were injected with saline before being confined to the vehicle-paired compartment for 30 minutes. Four hours later, mice were injected with 1.5 mg/kg of cocaine before being confined to the drug-paired compartment for 30 minutes. This phase lasted four days (4 sessions of conditioning with vehicle and 4 sessions of conditioning with cocaine). In the final phase (Post-C), the mouse was allowed to explore the box freely for 15 minutes, and the time spent in each compartment was recorded. The acquisition of CPP is indicated by a significantly higher time spent in the drug-paired compartment in Post-C compared to Pre-C. It is important to note that, as in our previous study (Martínez-Caballero et al., 2024), we used a low dose of cocaine (1.5 mg/kg) that was ineffective in naïve (non-stressed) mice in order to detect any potentiation of the rewarding effects of the drug in stressed animals. Regarding this, it is important to note that the procedure of CPP is characterized by a lack of dose-response effects. CPP is not a graded response, i.e., when a dose of cocaine is effective to induce CPP no greater effect can be detected with higher doses (Bardo and Bevins, 2000; Maldonado et al., 2006) or other manipulations that increases the rewarding effects of the drug (as the stress exposure).

**Results**

Ten mice were identified as outliers using the Z-scores method (Z=(X-mean)/standard deviation) in the more representative measurement of each behavioural test used. In particular, the Z-score values of mice deleted from the datasets employed to perform the ANOVAs were showed in Table S1. Each outlier mouse was eliminated only from the dataset of the particular behavioural measure in which it was an outlier.

Table S1. Z-score values of outlier mice.

**Social interaction test**

Figure S1. Short-term effects of VISD and VWR in the social interaction test. Bars represent mean (±SEM) values of the index of social interaction (ISI).

**Object recognition test**

All groups spent more time exploring the new object in comparison to the familiar object and have more frequency of exploration of the new object in comparison to the familiar one. The difference in the latency of exploration of both objects was only significant in mice of the group Control+VISD (see Table S2).

Table S2. Differences in the time exploring the objects (new and familiar), in the frequency of exploration and in the latency to explore the objects (Two-tailed paired Student t tests).

**References**

Arenas MC, Daza-Losada M, Vidal-Infer A, Aguilar MA, Miñarro J, Rodríguez-Arias M (2014) Capacity of novelty-induced locomotor activity and the hole-board test to predict sensitivity to the conditioned rewarding effects of cocaine. Physiol Behav 133: 152–160. doi: 10.1016/j.physbeh.2014. 05.028.

Campos AC, Fogaca MV, Aguiar DC, Guimarães FS (2013) Animal models of anxiety disorders and stress. Braz J Psychiatry 35: S101–S111. doi: 10.1590/1516-4446-2013-1139.

Henriques-Alves AM, Queiroz CM (2016) Ethological evaluation of the effects of social defeat stress in mice: beyond the social interaction ratio. Front Behav Neurosci 9:364. doi: 10.3389/fnbeh.2015.00364.

Krishnan V, Han MH, Graham DL, Berton O, Renthal W, Russo SJ (2007) Molecular adaptations underlying susceptibility and resistance to social defeat in brain reward regions. Cell 131: 391–404. doi: 10.1016/j.cell.2007. 09.018.

Maldonado C, Rodríguez-Arias M, Castillo A, Aguilar MA, Miñarro J (2007) Effect of memantine and CNQX in the acquisition, expression and reinstatement of cocaine-induced conditioned place preference. Prog. Neuropsychopharmacol. Biol Psychiatry 31: 932–939. doi: 10.1016/j.pnpbp. 2007.02.012.

Martínez-Caballero MÁ, Calpe-López C, García-Pardo MP, Arenas MC, de la Rubia Ortí JE, Bayona-Babiloni R, Aguilar MA (2024) Behavioural traits related with resilience or vulnerability to the development of cocaine-induced conditioned place preference after exposure of female mice to vicarious social defeat. Prog Neuropsychopharmacol Biol Psychiatry 130:110912. https://doi.org/10.1016/j.pnpbp.2023.110912

Montesinos J, Pascual M, Pla A, Maldonado C, Rodríguez-Arias M, Miñarro J, et al. (2015) TLR4 elimination prevents synaptic and myelin alterations and long-term cognitive dysfunctions in adolescent mice with intermittent ethanol treatment. Brain Behav Immun 45: 233-244. doi: 10.1016/j.bbi.2014.11.015.

Rodgers RJ, Johnson NJT (1995) Factor analysis of spatiotemporal and ethological measures in the murine plus-maze test of anxiety. Pharmacol Biochem Behav 52, 297–303. doi: 10.1016/0091-3057(95) 00138-m.

Smolinsky AN, Bergner CL, LaPorte JL, Kalueff AV (2009) Analysis of grooming behavior and its utility in studying animal stress, anxiety and depression. In:Gould T (ed) Mood and Anxiety Related Phenotypes in Mice. Neuromethods, vol 42 Humana Press, Totowa, pp 21–36. https://doi.org/10.1007/978-1-60761-303-9_2
